# High neuropeptide Y release associates with Ewing sarcoma bone dissemination - *in vivo* model of site-specific metastases

**DOI:** 10.18632/oncotarget.3345

**Published:** 2015-01-30

**Authors:** Sung-Hyeok Hong, Jason U. Tilan, Susana Galli, Ewa Izycka-Swieszewska, Taylor Polk, Meredith Horton, Akanksha Mahajan, David Christian, Shari Jenkins, Rachel Acree, Katherine Connors, Phuong Ledo, Congyi Lu, Yi-Chien Lee, Olga Rodriguez, Jeffrey A. Toretsky, Chris Albanese, Joanna Kitlinska

**Affiliations:** ^1^ Department of Oncology, Lombardi Comprehensive Cancer Center, Georgetown University Medical Center, Georgetown University, Washington DC, USA; ^2^ Department of Nursing, School of Nursing and Health Studies, Georgetown University, Washington DC, USA; ^3^ Department of Human Science, School of Nursing and Health Studies, Georgetown University, Washington DC, USA; ^4^ Department of Biochemistry and Molecular & Cellular Biology, Georgetown University Medical Center, Georgetown University, Washington DC, USA; ^5^ Department of Pathology and Neuropathology, Medical University of Gdańsk, Poland; ^6^ McGovern Institute, Massachusetts Institute of Technology, Boston, MA, USA; ^7^ Department of Pathology, Georgetown University Medical Center, Georgetown University, Washington DC, USA

**Keywords:** Ewing sarcoma, neuropeptide Y, bone invasion, animal model

## Abstract

Ewing sarcoma (ES) develops in bones or soft tissues of children and adolescents. The presence of bone metastases is one of the most adverse prognostic factors, yet the mechanisms governing their formation remain unclear. As a transcriptional target of EWS-FLI1, the fusion protein driving ES transformation, neuropeptide Y (NPY) is highly expressed and released from ES tumors. Hypoxia up-regulates NPY and activates its pro-metastatic functions. To test the impact of NPY on ES metastatic pattern, ES cell lines, SK-ES1 and TC71, with high and low peptide release, respectively, were used in an orthotopic xenograft model. ES cells were injected into gastrocnemius muscles of SCID/beige mice, the primary tumors excised, and mice monitored for the presence of metastases. SK-ES1 xenografts resulted in thoracic extra-osseous metastases (67%) and dissemination to bone (50%) and brain (25%), while TC71 tumors metastasized to the lungs (70%). Bone dissemination in SK-ES1 xenografts associated with increased NPY expression in bone metastases and its accumulation in bone invasion areas. The genetic silencing of NPY in SK-ES1 cells reduced bone degradation. Our study supports the role for NPY in ES bone invasion and provides new models for identifying pathways driving ES metastases to specific niches and testing anti-metastatic therapeutics.

## INTRODUCTION

Ewing sarcoma (ES) is a malignant tumor that arises in bones or soft tissues of children and adolescents. While there has been significant improvement in the outcome of patients with localized ES, the treatment of disseminated disease remains an unsolved clinical problem, with a 3-year event-free survival (EFS) of 27% for patients with metastatic ES [1, 2]. The localization of metastases is an important prognostic factor. While lung metastases are the most common, the survival is worse for patients with secondary dissemination to bone, particularly when both bone and pulmonary metastases are present (8-14% EFS), and dismal for rare cases with brain metastases (2.7 months median survival) [1, 3]. Currently, patients with metastatic ES are treated with the same standard therapy as those with localized disease. This lack of adequate and specific therapies for metastatic ES reflects a poor understanding of the mechanisms governing ES dissemination. Studies aimed at identifying such mechanisms are hindered by the limited availability of clinical material and lack of animal models that accurately recapitulate metastatic processes. The majority of previous metastatic models involve intravenous tumor cell injections, which omit the initial steps of the tumor cell establishment and migration [4-9]. Moreover, the analyses often focus solely on lung metastases, thus not advancing mechanistic understanding of tumor metastasis to other locations, including bone [5, 9-13].

Malignant transformation of ES is driven by chromosomal translocations resulting in the fusion of the EWS gene with the ETS transcription factor, the most frequent being t(11;22)(q24;q12) giving rise to EWS-FLI1 protein [14]. There are several known variants of the EWS-FLI1 transcript, which vary in their fusion sites and transactivation potencies [15]. Microarray analyses have identified neuropeptide Y (NPY) as an EWS-FLI1 transcriptional target that is highly expressed in ES cells and released from ES tumors [16-20]. NPY, acting via its multiple G protein-coupled receptors designated Y1R–Y5R, is known to regulate proliferation, survival and differentiation of a variety of cells [21-28]. Our previous studies revealed pleiotropic and opposing actions of NPY in ES ranging from Y1R/Y5R-mediated tumor cell death to Y2R/Y5R-mediated angiogenesis and ES cancer stem cell proliferation and migration [18-20]. We have also shown that a hypoxic tumor microenvironment enhances pro-metastatic actions of the peptide [20]. Furthermore, our clinical data revealed a trend toward elevated NPY release in ES patients with metastatic tumors, as well as significantly higher systemic NPY levels in pelvic and axial ES of bone origin [29]. The latter observation correlates with the previously reported role of NPY in regulation of bone homeostasis and implicates its involvement in ES bone invasion [24, 30-32].

Given the multifaceted actions of NPY in ES and its hypoxia-inducible pro-metastatic actions, we sought to determine if high release of the peptide contributes to ES dissemination and promotes colonization of particular metastatic niches, mimicking the phenotype observed in patients. To this end, we developed an *in vivo* metastatic model that recapitulates all stages of the ES metastatic process, starting from primary tumor growth and resection, through local invasion, and formation of distant metastases [10]. Using this approach we observed a high frequency of distant bone metastases in ES tumors that secrete a significant amount of NPY, while ES xenografts with low NPY expression and release metastasized exclusively to lungs. Moreover, the extent of local bone invasion in primary tumors correlated with NPY levels and was reduced by the genetic silencing of NPY. These results support a potential role of NPY in ES bone invasion. Furthermore, our orthotopic xenograft models can be used as a platform for studying site-specific ES metastases, providing an opportunity to investigate the mechanisms of tumor dissemination to particular niches and test novel therapeutic approaches targeting such pathways. This model is of particular value for investigation of bone metastases, which are difficult to model in experimental setting and carry the worse prognosis.

## RESULTS

### ES cell lines, TC71 and SK-ES1, differ in NPY release

**Figure 1:**
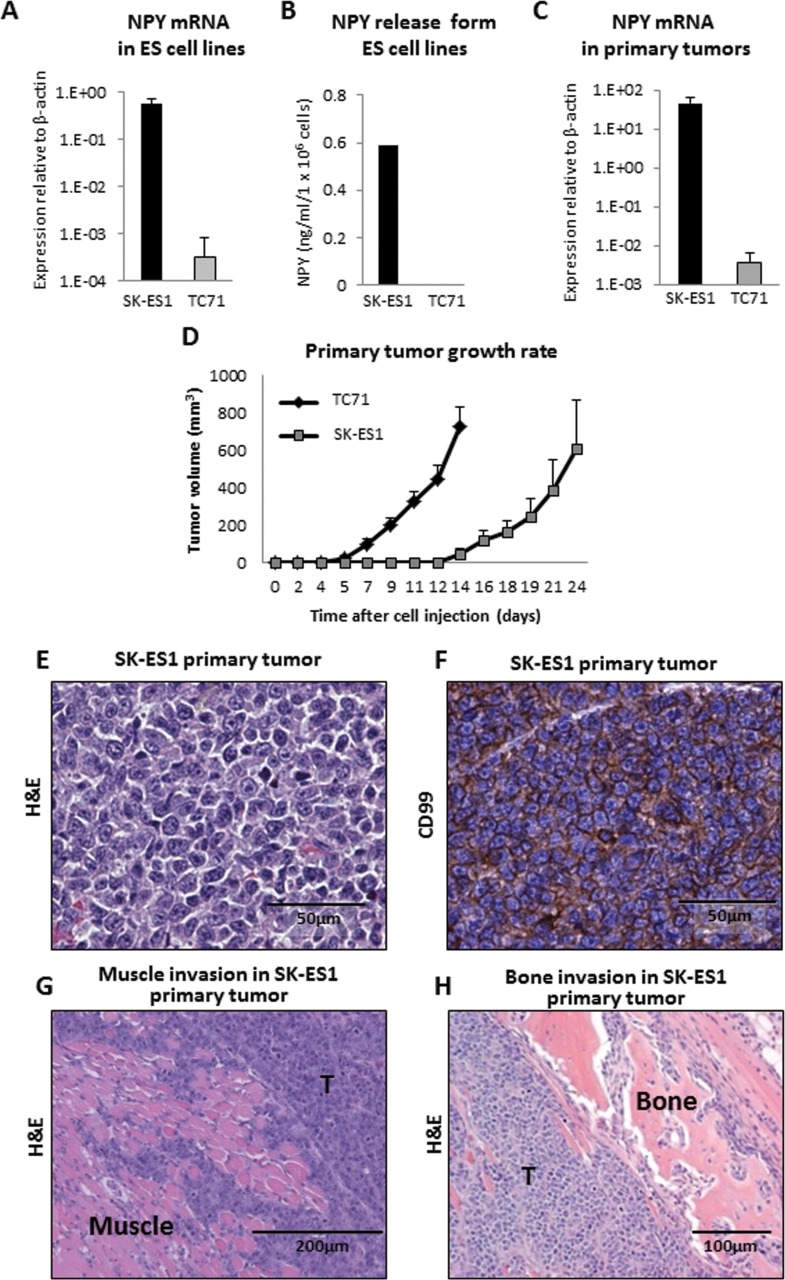
TC71 and SK-ES1 ES cells varying in NPY expression and release give rise to invasive primary tumors A. NPY mRNA levels measured by real-time RT-PCR in SK-ES1 and TC71 cells cultured *in vitro*. B. NPY concentrations in conditioned media from SK-ES1 and TC71 cells measured by ELISA. C. TC71 and SK-ES1 cells were injected into gastrocnemius muscles of SCID/bg mice. NPY mRNA levels in primary tumors were measured by real-time RT-PCR. D. The growth of TC71 and SK-ES1 primary tumors was monitored by periodical measurements of the tumor-bearing hindlimb. E. Primary tumor mass of SK-ES1 xenograft. F. Positive staining of SK-ES1 primary tumor for the ES marker, CD99. G. Muscle invasion within SK-ES1 primary tumor. H. Bone degradation within SK-ES1 primary tumor. Panels E-H contain representative photographs of the SK-ES1 primary tumors. However, tumor morphology was similar in SK-ES1 and TC71 xenografts. T - tumor; H&E – hematoxylin and eosin staining.

As an EWS-FLI1 target, NPY is universally expressed in ES [18-20]. However, ES cell lines vary significantly in their levels of NPY expression and release. To determine if high NPY secretion influences the pattern of metastases, we used two ES cell lines, SK-ES1 and TC71, which express high and low NPY levels, respectively (Fig. 1A). These differences in expression of the peptide translated to a variability in its release. Conditioned media from SK-ES1 cells contained high levels of NPY (average of 0.6 ng/ml/10^6^ cells), while no secretion to the media was observed in TC71 cells (Fig. 1B).

### *In vivo* orhotopic xenograft model of metastatic ES

To compare the metastatic potential and pattern of disease dissemination between ES cell lines that express different NPY levels, we developed an animal model which closely recapitulates the disease progression in ES patients. ES cells were injected into gastrocnemius muscles of SCID/beige mice, and the tumors were allowed to grow. Once primary tumors reached a volume of 1 cm^3^, they were surgically resected to reduce morbidity associated with excessive tumor burden and to allow metastases to form. Progression of the disease was monitored by MRI. Importantly, differences in NPY expression observed *in vitro* between SK-ES1 and TC71 cell lines were preserved *in vivo*, within primary tumor tissues (Fig. 1C).

### Primary tumor growth

For both ES cell lines, the frequency of primary tumor growth was 100%. TC71 primary tumors exhibited faster growth rate and lower variability in overall size, as compared to those derived from SK-ES1 cells (Fig. 1D). However, both cell lines produced large tumors that were consistent in morphology with human ES and positive for the ES marker, CD99 (Fig. 1E, F). Moreover, in all cases tested, marked local muscle invasion and bone degradation within the primary tumor was observed (Fig. 1G, H).

### TC71 xenografts metastasize to the lungs

**Figure 2:**
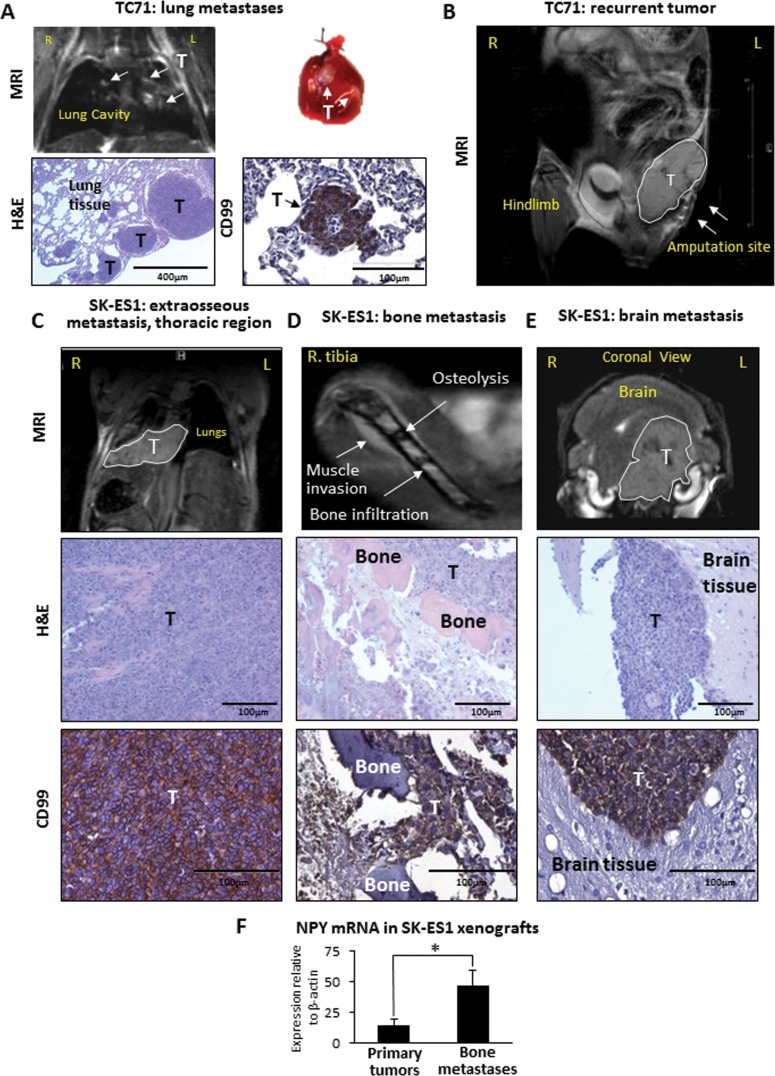
TC71 xenografts give rise to lung metastases and local relapses, while SK-ES1 xenografts metastasize to soft tissues of the thoracic region, bones and brain A. TC71 pulmonary metastases detected by magnetic resonance imaging (MRI) and macroscopic observation. The presence of metastases was confirmed by histological examination and positive staining for ES marker, CD99. B. MRI of recurrent TC71 tumor (white outline) adjacent to the amputation site (arrows). C. Soft tissue metastasis in thoracic region of mouse bearing an SK-ES1 xenograft. The tumor below lungs is identified by MRI (white outline), histopathology and positive staining for the ES marker, CD99. D. Distant metastasis to the contralateral tibia detected by MRI. Histopathology analysis confirms massive bone degradation within tumor. The presence of tumor cells as confirmed by CD99 staining. E. MRI of SK-ES1 metastasis to the brain (white outline) confirmed by histopathology and CD99 immunohistochemistry. F. NPY mRNA in SK-ES1 primary tumors and corresponding distant bone metastases measured by real-time RT-PCR. R – right; L – left; T – tumor; H&E – hematoxylin and eosin staining; * - p<0.05.

Consistent with the rapid growth of primary tumors, progression of the disease to metastasis in mice bearing TC71 xenografts was observed within 2-3 weeks (average of 18 days post-amputation to the first metastasis detection). The tumors metastasized exclusively to the lungs (70% of cases) and no other distant metastases were detected (Fig. 2A, Table 1). However, 90% of mice bearing TC71 xenografts developed local relapses in areas adjacent to the amputation sites, which gave rise to abdominal tumors (Fig. 2B), suggesting a high local invasiveness of TC71 cells.

**Table 1:** Comparison of metastatic pattern in SK-ES1 and TC71 orthotopic xenografts

Type of metastases:	SK-ES1	TC71	p value SK-ES1 vs TC71
	**Days**		
Average number of days post amputation to first metastasis detection	38	18	< 0.001
Average survival days post-amputation	45	28	< 0.001
Average survival days from cell injection	76	45	< 0.001
	**% of cases (number of positive cases/total mouse number)**		
Any distant metastasis	92% (11/12)	70% (7/10)	NS
Soft tissue metastases	67% (8/12)	0% (0/10)	< 0.01
Bone metastases (limb, jaw, spine)	50% (6/12)	0% (0/10)	< 0.01
Lung metastases	0% (0/12)	70% (7/10)	< 0.001
Brain metastases	25% (3/12)	0% (0/12)	NS
Recurrent tumors	17% (2/12)	90% (9/10)	< 0.001

### SK-ES1 xenografts metastasize to bones and soft tissues, but do not develop pulmonary metastases

Progression of the disease in mice bearing SK-ES1-xenografts was less rapid than upon injections with TC71 cells (average of 38 days post-amputation to first metastasis detection) and characterized by a completely distinct pattern of metastases (Table 1). 67% of these mice presented with rapidly growing soft tissue metastases within the thoracic region (Fig. 2C). In one such case, the local invasion of the thoracic tumor into the lung tissue was observed. 50% of animals had distant bone metastases, as confirmed by histopathology and MRI (Fig. 2D). The affected bones included jaw, ribs, vertebra, contralateral tibia. In addition, three out of twelve mice had brain metastases detectable by MRI (Fig. 2E). However, no established hematogenous pulmonary metastases were observed. Lastly, only two animals presented with recurrent tumors in areas adjacent to the amputation sites, which is an indicator of lower local invasiveness of SK-ES1 cells *in vivo*. The dissemination of SK-ES1 cells to bone was associated with elevated expression of NPY in bone metastases, as compared to their corresponding primary tumors (Fig. 2F). No statistically significant increase in NPY expression was observed in SK-ES1 soft tissue metastases.

### Cells derived from distant metastases preserve an enhanced pro-metastatic phenotype

**Figure 3:**
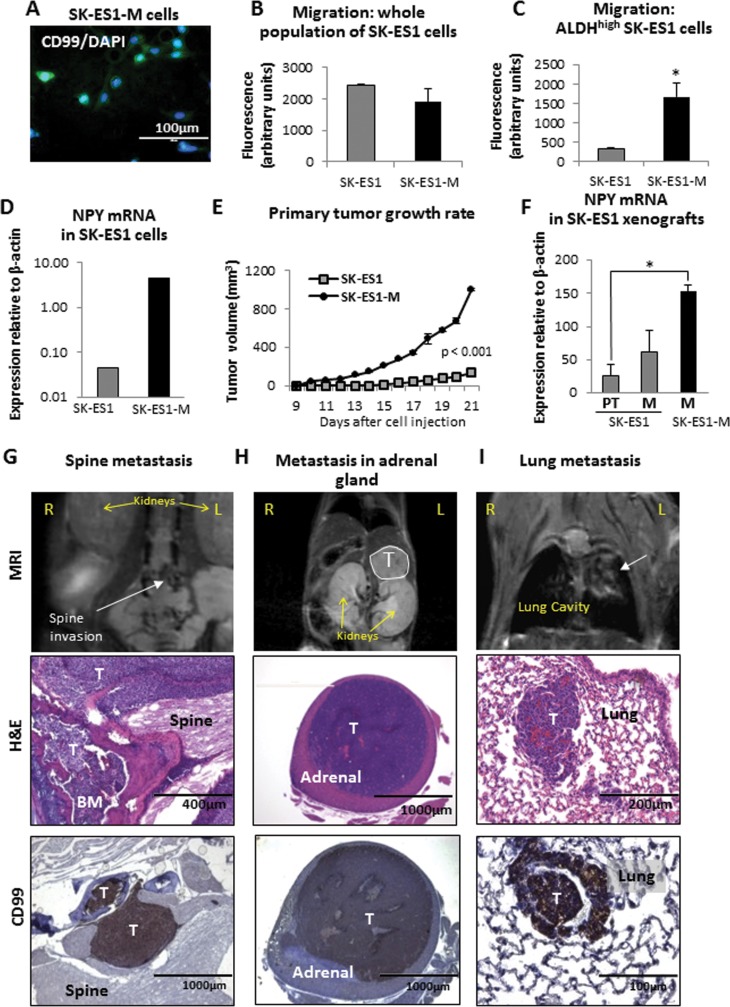
Cells derived from SK-ES1 metastases, SK-ES1-M, exhibit increased motility in the cancer stem cell fraction, enhanced growth *in vivo* and metastatic potential A. SK-ES1-M cells were isolated from SK-ES1 metastasis derived from the orthotopic xenograft model. The cells were immunostained for the ES marker, CD99, and couterstained with DNA-binding fluorescent stain, DAPI. All DAPI-stained cells are also positive for CD99. B. Migration of the original SK-ES1 cells and metastases-derived SK-ES1-M cells, assayed as the whole cell population using BD FluoroBlok™ 96-well plates. C. Migration of FACS-sorted ALDH^high^ subpopulation of SK-ES1 and SK-ES1-M cells, measured as above. D. NPY mRNA levels in original SK-ES1 and SK-ES1-M cells measured by real time RT-PCR. E. SK-ES1 and SK-ES1-M cells were injected into gastrocnemius muscles of SCID/bg mice. Volumes of the primary tumors were assessed by longitudinal measurements of the tumor-bearing lower extremity, and the growth rates were compared between the two cell lines. F. NPY mRNA in tissues from SK-ES1 and SK-ES1-M primary tumors (PT) and metastases (M) measured by real time RT-PCR G. The SK-ES1-M xenograft-derived metastasis to the spine detected by MRI (white arrow), histopathology and positive CD99 staining. H. SK-ES1-M metastasis to adrenal gland (MRI, white outline). I. Lung metastasis (MRI, white arrow) derived from SK-ES1-M xenograft. R – right; L – left; T – tumor; BM – bone marrow; H&E – hematoxylin and eosin staining; * - p<0.05.

Differences in gene expression between SK-ES1 primary tumors and metastases suggested that disease dissemination involves acquisition of a specific phenotype, which facilitates colonization of metastatic niches. To prove this, we isolated and characterized cells from both primary tumors and distant metastases derived from our orthotopic model. The cells were 100% positive for ES marker, CD99 (Fig. 3A), confirming tumoral origin of the cells. Interestingly, there were no significant differences in either cell migration (Fig. 3B) or low attachement growth (data not shown) between the original cells and those derived from metastases (SK-ES1-M), when the cells were tested as a whole population. However, a significant increase in motility was observed in SK-ES1-M cells, when the populations with high activity of aldehyde dehydrogenase (ALDH^high^) selected from SK-ES1 and SK-ES1-M cell lines were assayed (Fig. 3C). Such ALDH^high^ cells were previously characterized as ES tumor initiating cells [33]. SK-ES1-M cells also had increased NPY mRNA levels (Fig. 3D) and upon re-introduction to the animals exhibited a significant increase in tumor growth rate as compared to the original SK-ES1 cell line (Fig. 3E). Strikingly, metastases derived from SK-ES1-M cells had markedly elevated NPY mRNA levels, as compared to both the primary tumors and the metastases from which they were derived (Fig. 3F). This was associated with augmented metastatic potential of SK-ES1-M xenografts, as evidenced by an increase in the number of metastases and in the range of affected organs. The average number of distant metastases per mouse was 5.5 for animals injected with SK-ES1-M versus an average of 2 distant metastases for animals bearing SK-ES1 xenografts. SK-ES1-M metastases were detected in soft tissues of the thoracic region and bones (rib, vertebra), as observed in the original cell line, but also in other organs, including spine, adrenal glands and ovaries (Fig. 3G, H). SK-ES1-M xenografts also gave rise to lung metastases, which were not observed in mice injected with the original SK-ES1 cells (Fig. 3I). Thus, cells derived from SK-ES1 metastases were capable of inducing rapidly progressing, wide spread systemic disease upon reintroduction to the animals.

### NPY accumulates in areas of bone invasion

**Figure 4:**
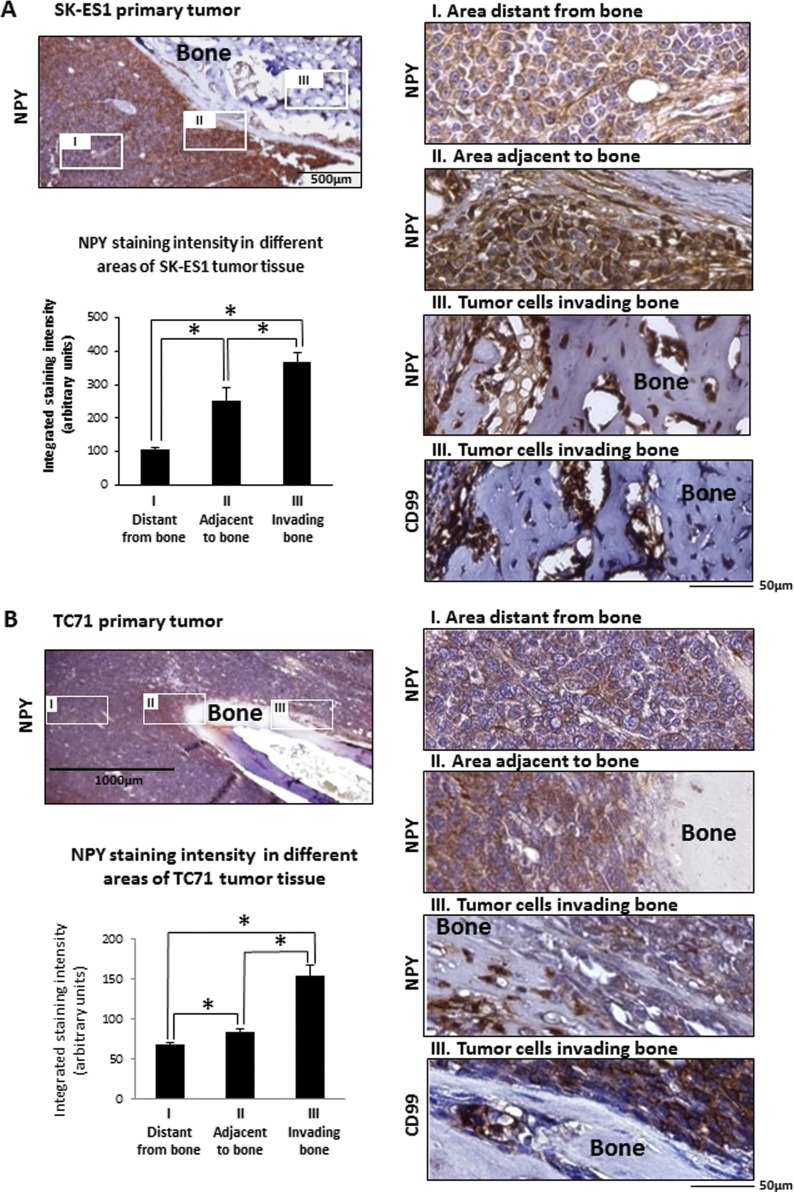
Induction of NPY expression in areas of bone invasion within ES primary tumors A. NPY immunostaining of SK-ES1 primary tumor. Representative low magnification photograph of the tumor area adjacent to the bone and higher magnification images of specific areas: I - tumor tissue distant from the bone; II – tumor tissue directly surrounding bone and III - tumor cells invading the bone tissue. CD99 staining identifies groups of cells within bone tissue as tumor cells. Quantification of NPY staining intensities within different areas of primary tumors was performed using ImageJ software. B. NPY and CD99 immunostaining in TC71 primary tumor analyzed and quantified as above; * - p<0.05.

The high frequency of dissemination to bone in NPY-rich SK-ES1 xenografts and elevated expression of the peptide in bone metastases suggested a potential contribution of NPY to ES bone invasion. To investigate this, we compared patterns of NPY immunostaining in SK-ES1 and TC71 primary tumors. While strong NPY immunostaining was observed across the entire SK-ES1 xenograft tissue, its intensity was significantly higher in tumor tissue adjacent to the bone, as compared to regions distant from the bone invasion area (Fig. 4A). Moreover, the most intense NPY immunostaining among all cell fractions tested was seen in groups of CD99-positive tumor cells invading the bone. As expected, based on the low NPY mRNA levels, TC71 primary tumors presented with weak NPY immunoreactivity (Fig. 4B). However, similar to that seen with SK-ES1 cells, NPY immunostaining was significantly elevated in areas adjacent to the bone and was the highest in cells directly invading the bone tissue. These observations suggested induction of NPY expression in TC71 tumors by bone-derived factors.

### Inhibition of NPY expression reduces tumor-induced bone destruction

**Figure 5:**
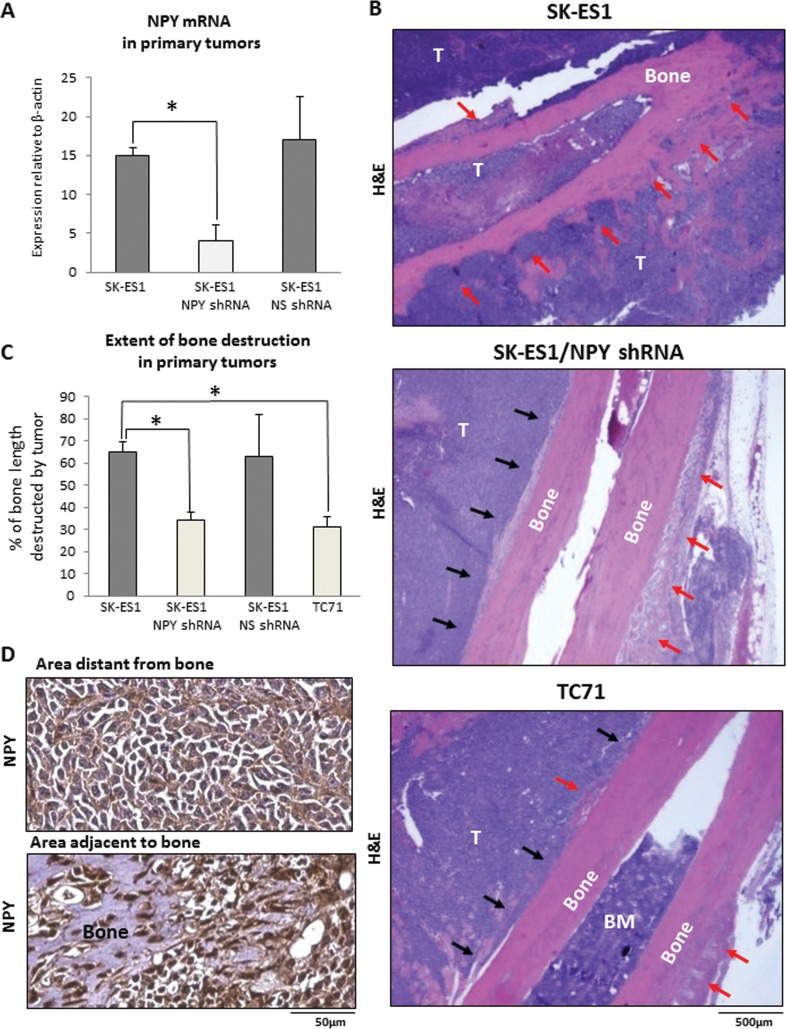
NPY knock-down reduces bone degradation in SK-ES1 primary tumors A. Efficiency of NPY shRNA knock-down in SK-ES1 cells measured by real time RT-PCR. B. Representative images of hematoxylin and eosin stained longitudinal bone cross-sections from SK-ES1, SK-ES1/NPY shRNA and TC71 primary tumors. Black and red arrows indicate intact and degraded bone surfaces, respectively. C. Bone destruction index calculated for SK-ES1, SK-ES1/NPY shRNA, SK-ES1/NS shRNA and TC71 primary tumors as the percent of the bone length with tumor-induced degradation. D. NPY immunostaining in areas distant and adjacent to the bone in SK-ES1/NPY shRNA primary tumors. NS shRNA - non-silencing shRNA; T – tumor; BM – bone marrow; H&E – hematoxylin and eosin staining; * - p<0.05.

To directly test the role of NPY in bone invasion, we compared the extent of bone destruction in primary tumors derived from original SK-ES1 cells and their counterparts stably expressing NPY shRNA (SK-ES1/NPY shRNA). NPY shRNA reduced NPY mRNA levels in SK-ES1 cells by 75% (Fig. 5A). The down-regulation of NPY expression inhibited local bone destruction in primary tumors, as manifested by the presence of extended segments of intact bone surface adjacent to the tumor tissue (Fig. 5B). Consequently, the bone destruction index measured as a percent of bone length with detectable bone tissue abnormalities was significantly reduced in SK-ES1/NPYshRNA primary tumors, as compared to tumors derived from the original SK-ES1 cells, and comparable to that observed in TC71 xenografts naturally expressing low levels of the peptide (Fig. 5B, C). Notably, despite the overall decreased intensity of NPY immunostaining in SK-ES1/NPYshRNA tumors, expression of the peptide was induced in areas adjacent to the bone with apparent signs of destruction, and in cells invading bone tissue, as was previously observed (Fig. 5D). No decrease in bone destruction index was detected in xenografts derived from SK-ES1 cells transduced with control vector expressing non-silencing shRNA (SK-ES1/NS shRNA) (Fig. 5C).

## DISCUSSION

The presence of distant bone metastases associates with the most adverse prognosis in ES [1, 2]. Even though some factors contributing to their formation, such as Dickkopf-related protein 2 (DKK2), have been identified, the detailed mechanisms promoting their development remain unclear [5, 34]. This, in turn, results in a lack of adequate therapies for patients at this stage of the disease. Studies aimed at the identification of novel therapeutic targets are hindered by the limited availability of clinical samples from ES metastases. Thus, creating appropriate animal models is of the utmost importance for our understanding and ability to target bone dissemination in ES patients. Here we report that using NPY-rich ES cells in an orthotopic xenograft model results in frequent and robust distant bone metastases. Importantly, our model recapitulates all the steps of disease progression observed in ES patients. We were able to mimic the growth of primary tumors in their natural orthotopic environment, including local invasion to bone, muscles and other soft tissues, followed by its resection and development of distant metastases. Although a similar approach has been previously applied to TC71 and EWS-925 cell lines in other studies, the metastatic pattern has not been described in detail and the analyses were focused solely on lung metastases [10]. The differential metastatic pattern observed in our study – high frequency of distant bone metastases in NPY-rich SK-ES1 xenografts and pulmonary dissemination of TC71 tumors – underscores differences in the biology of these metastases and provides an opportunity to pinpoint the specific mechanisms governing their formation. Notably, primary cells derived from such distant metastases preserve their metastatic potential, as evidenced by their enhanced growth and dissemination *in vivo*. Thereby, this cellular model may become a valuable experimental tool to identify novel metastatic pathways and therapeutic targets that can be subsequently validated in our ES animal model. Further studies are required to determine if metastasis-derived cells will also preserve the specificity of the niches they colonize.

Although formation of distant bone metastases has been previously reported using other cell lines and animal models, the 50% frequency of their development observed in SK-ES1 orthotopic xenografts is one of the highest described [4, 6-8, 10, 35]. Dissemination to bone was also shown for TC71 cells after tail vein injection or intrafemoral transplantation [4, 6-8, 35]. However, systemic injections of ES cells bypass the initial steps of the metastatic process and result in an inconsistent frequency of bone metastases, often as low as 16-25% [4, 6-8]. The intrafemoral injection of TC71 cells leads to development of distant bone metastases in 27% of mice [35]. The discrepancy between a lack of TC71 bone metastases in our model and their formation in other models may result from differences in the route of cell administration. It is plausible that direct exposure of tumor cells to the bone environment, e.g. upon their direct injection to the bone cavity, induces changes promoting their subsequent dissemination to other bones. This notion is supported by the specific pattern of NPY immmunostaining observed in TC71 primary tumors. Despite the overall low NPY immunoreactivity in the tumor mass, the peptide expression was elevated in tumor cells adjacent to bone invasion areas and directly invading the bone. A similar gradient of NPY expression was also observed in SK-ES1 and SK-ES1/NPY shRNA primary tumors, suggesting that bone-derived factors can change the biology of tumor cells. Such influence of the bone microenvironment on tumor cells has been previously described as the “vicious cycle” of bone metastases. The initial bone destruction leads to release of growth factors residing in the bone matrix, such as transforming growth factor β (TGF-β) [36]. These factors then stimulate tumors cells to further increase the release of osteolytic molecules. Moreover, the high ambient calcium concentration present during bone resorption or stiffness of the bone tissue may change tumor cell properties [36]. In the case of NPY, such an increase in its expression may be driven by brain-derived neurotrophic factor (BDNF), which is abundant in the bone environment and has been shown to induce NPY expression and release [37, 38]. In our model the local bone invasion and interaction of tumor cells with bone components is a secondary event, which provides a plausible explanation for the predominance of bone environment-independent pulmonary metastases in mice bearing TC71 xenografts. In contrast, SK-ES1 cells constitutively express and release high levels of NPY, and perhaps other factors promoting bone invasion, which results in their frequent and rapid dissemination to distant bones.

Preferential metastases to bone have been previously shown for cells transduced with type 2 and 3 EWS-FLI1 fusion proteins, but not with the type 1 transgene [15]. In line with this, SK-ES1 cells with high affinity to bone carry the type 2 EWS-FLI1 translocation, while TC71 cells have the type 1 fusion. Interestingly, we have recently shown that ES cell lines carrying type 2 and 3 fusions, such as SK-ES1, RDES, MMHES1 and A4573, secrete high NPY levels, while no NPY was detected in the media of cells with type 1 EWS-FLI1 [29]. On the other hand, however, no significant differences in the serum concentration of NPY in ES patients with various translocation types were observed, indicating that the systemic levels of the peptide could be further modified by other clinical variables, such as tumor size and localization [29]. Thus, while the translocation type may be responsible for differential expression of anti-osteogenic and osteolytic factors, including NPY, their levels can be further modified in the tumor microenvironment, as suggested by our current and previous data [20].

Our previous *in vitro* studies implicate NPY as a hypoxia-inducible pro-metastatic factor. We have shown that hypoxic conditions activate Y2R/Y5R-mediated actions of NPY, which stimulate tumor vascularization and promote ES cell proliferation and migration, the processes known to facilitate tumor dissemination [20]. Strikingly, the mitogenic and pro-migratory effects of NPY are limited to a population of ALDH^high^ ES tumor initiating cells, which correlates with increased motility in this cell fraction isolated from SK-ES1-M cells in the current study [20, 33]. These results, along with elevated NPY expression in metastatic SK-ES1-M cells, implicate the peptide as a candidate mediator of the increase in their metastatic potential. In line with this, we have recently shown that high NPY expression in ES tumors results in its secretion to the circulation and elevated systemic levels of the peptide in ES patients [29]. Serum NPY concentrations are particularly high in patients with metastatic ES and significantly elevated in those with tumors of bone origin, while NPY mRNA levels are increased in bone ES, as compared to extra-osseous lesions. Thus, the ES phenotype associated with high NPY release in the human population correlates well with the metastatic pattern observed in our ES orthotopic xenograft mouse model. High frequency of distant bone metastases in NPY–rich SK-ES1 xenografts, but not in mice injected with TC71 cells, which do not release NPY under basal conditions, corroborates the role of the peptide in promoting bone metastases. This notion is further supported by the reduced bone destruction in SK-ES1/NPY shRNA and TC71 primary tumors, as compared to SK-ES1 xenografts, as well as the accumulation of NPY-rich cells in front of local bone invasion areas. Of note, no bone metastases and any distant tumor dissemination was also observed in mice injected with another ES cell line characterized by low NPY release, SK-N-MC (data not shown) [19, 29].

The experimental and clinical evidence supporting the potential role of NPY in ES bone invasion is in agreement with previous reports indicating its role in the regulation of bone homeostasis. The peptide has been shown to inhibit osteoblast differentiation and bone formation [30, 32]. The effect of NPY is potent enough to trigger changes in skeletal development, as evidenced by increased bone density in NPY knockout mice [39]. Although ES bone lesions are mostly osteoclastic, these tumors develop predominantly in children and adolescents with ongoing bone formation that requires a tight regulation of both osteoblastic and osteoclastic activity. Therefore, disrupting proper osteoblast differentiation and bone formation by tumor-derived NPY may facilitate bone degradation via shifting the balance toward osteolysis. Similarly, the crucial role of factors blocking osteoblast differentiation, such as the Wnt pathway inhibitor, DKK-1, in bone invasion has been shown for neuroblastoma, another pediatric tumor with frequent bone metastases [40]. In addition to its direct effects on osteoblasts, NPY has been shown to regulate balance between two major osteoblast-derived regulators of osteolytic activity - nuclear factor kappa-B ligand (RANKL) and osteoprotegerin (OPG) [41]. Lastly, NPY induces release of interleukin 6 (IL-6) from macrophages [42]. IL-6 is an established osteolytic factor, promoting bone resorption by a variety of mechanisms, including direct activation of osteoclasts and stimulation of osteolytic factor release from tumor and stromal cells within bone tissue [43, 44]. Thus, NPY can also affect metastatic processes indirectly by inducing changes in the bone environment. Altogether, our current findings and previous evidence of anti-osteogenic activity of NPY warrant further investigation into its role in ES bone dissemination and the mechanisms of actions.

Aside from bone metastases, SK-ES1 xenografts presented with frequent soft tissue metastases in the thoracic region. Interestingly, this tumor localization, along with local rib and lung invasion observed in our model, is a characteristic of Askin tumors - a specific subgroup of primitive neuroectodermal tumors (PNET) [45, 46]. This thoracopulmonary localization of SK-ES1 metastases implicates specific biological features of tumor cells, which facilitate their growth in this particular region [45, 46]. Another characteristic feature of SK-ES1 tumors is their affinity for neural tissues, as manifested by the presence of brain and spine metastases. To our knowledge, this is the first report of central nervous system metastases in an animal model of ES. Although rare, brain metastases are associated with a particularly poor outcome in ES patients and factors facilitating their formation remain unknown [3]. Given the unfavorable prognosis of Askin tumors (14% 6-year survival) and ES patients with secondary cerebral tumors, uncovering the mechanisms underlying such properties of ES subsets via our model may inform novel therapeutic approaches [45, 46].

In summary, using ES cell lines with differential NPY expression in an orthotopic xenograft metastatic mouse model provides an opportunity to investigate molecular events governing ES dissemination to specific metastatic niches and test anti-metastatic therapies. Importantly, our model recapitulates all stages of ES metastases and mimics the metastatic patterns observed in ES patients, which confirms its clinical relevance. In particular, orthotopic xenografts derived from NPY-rich ES cells serve as an invaluable tool to investigate bone and CNS distant metastases, which are associated with the most adverse prognosis in ES patients. Furthermore, our findings support the role of NPY in the biology of ES. We provide direct evidence for the role of NPY in tumor-induced bone degradation and an insight into its potential involvement in ES dissemination. Understanding NPY actions in ES may also have implications for other malignancies rich in NPY and its receptors, such as neuroblastoma and breast cancer, both of which are known to metastasize to bones.

## MATERIALS AND METHODS

### Cell culture

Human ES cell lines, SK-ES1 and TC71, were obtained and cultured as previously reported [19]. For primary culture, tissue samples from SK-ES1 primary tumors and metastases were transferred into a 10 cm cell culture plate, cut into pieces and cultured in RPMI media (ATCC, Manassas, VA) supplemented with 10% FBS. After a few days, cellular outgrowth from the tissue pieces was observed. Once cells reached confluence, they were trypsinized and propagated according to standard cell culture techniques. SK-ES1 cells stably expressing NPY shRNA (SK-ES1/NPY shRNA) or non-silencing shRNA (SK-ES1/NS shRNA) were created by transduction of SK-ES1 cells with a pGIPZ lentiviral vector (Thermo Scientific, Waltham, MA) encoding the corresponding shRNAs followed by puromycin selection.

### Real time RT-PCR

RNA from cultured cells was isolated using High Pure RNA Isolation Kit (Roche Applied Science, Indianapollis, IN) and from tissues using TRI reagent (Sigma, St. Louis, MO). cDNA was synthesized using iScript cDNA Synthesis Kit and amplified using ICycler iQ Detection System (Bio-Rad Laboratories, Hercules, CA), TaqMan Universal PCR Master Mix and pre-designed primers and fluorescein-labeled probes (Applied Biosystems, Foster City, CA). The results were calculated by the comparative CT method using β-actin as a reference gene.

### ELISA

Conditioned media were collected from ES cells upon 24h culture, and cells were trypsinized and counted. NPY concentration in culture media was determined using the Neuropeptide Y Enzyme Immunoassay Kit (Bachem Peninsula Laboratories, San Carlos, CA) and normalized per cell number.

### *In vivo* model

2 × 10^6^ of ES cells suspended in 0.1 ml of PBS were injected into gastrocnemius muscles of 4-6 weeks old female SCID/beige mice [10]. The growth of primary tumors was monitored by periodical measurements of the limb and the volume of tumors calculated according to the formula (D x d2/6) x π, where D was the longer diameter and d was the shorter diameter. Once tumors reached a volume of 1cm^3^, tumor-bearing limbs were amputated. Metastasis formation was monitored by periodic magnetic resonance imaging (MRI). Once distant metastases were detected, the mice were euthanized. Macroscopic metastases and organs were harvested and fixed in 10% buffered formalin for histopathological analyses. All procedures were approved by the Georgetown University Institutional Animal Care and Use Committee.

### Imaging

MRI was performed at the Preclinical Imaging Research Laboratory at Georgetown University Medical Center on a 7 Tesla Bruker horizontal spectrometer run by Paravision 5.1 software, as previously described [47-51]. During imaging the mice were anesthesized with 2% isoflurane, 30% oxygen and 70% nitrous oxide, placed on a holder with respiration monitorization and imaged either in a 40 or 23 mm Bruker mouse volume coil for whole body and brain imaging, respectively. The anatomical imaging protocols used were two-dimensional, T2-weighted RARE sequences that included the following parameters: TR = 3000 ms, TE = 24 ms, matrix = 256 × 256, FOV = 4.35 × 3.0 cm, slice thickness = 0.5 mm, RARE factor = 4 and averages = 4.

### Histopathology and immunohistochemistry

Hematoxylin and eosin stained tissue sections were assessed by pathologists – Drs. Galli and Izycka-Swieszewska. The bone destruction index was calculated as a percent of total bone length with detectable signs of bone tissue abnormalities including areas of tumor invasion, osteolytic and osteoblastic changes, and measured using ImageJ software (National Institutes of Health, Bethesda, MD) [52]. Immunostaining was performed on formalin-fixed, paraffin-embedded tissues using rabbit polyclonal anti-NPY antibody (Sigma, St. Louis, MO) and monoclonal mouse anti-CD99 antibody (DAKO, Carpinteria, CA). The same anti-CD99 antibody was used for immunostaining of paraformaldehyde-fixed cells cultured *in vitro*, followed by detection with FITC-conjugated anti-mouse antibody (Life Technologies, Grand Island, NY). NPY immunostaining was quantified as an integrated staining intensity using NIH Scion Image software (Scion Corp., Frederick, MD). Direct comparisons of staining intensities between different cell fractions were made within one section.

### Aldefluor assay and cell sorting

ES cells were stained using the ALDEFLUOR kit (Stemcell Technologies, Vancouver, Canada). Fluorescence-activated cell sorting (FACS) was performed on FACSAria (BD, Franklin Lakes, NJ), utilizing FACSDiva and FCS Express 4 softwares (DeNovo Software, Los Angeles, CA). The ALDH^high^ cell fraction was selected by sorting for the upper 8% of cells, as previously described [20].

### ES cell migration

ES cells, either the entire population or FACS-sorted ALDH^high^ cells, were plated onto BD FluoroBlok™ 96-well plates (BD Biosciences, San Jose, CA) with serum free media in the upper chamber and media supplemented with 5% FBS in the lower chamber. The cells were then incubated for 18 h and stained with Calcein AM. Cell migration was quantified based on fluorescence measured from the bottom of the plate.

### Statistical analyses

Statistical analyses were performed using GraphPad software (GraphPad Software, Inc; La Jolla, CA). The comparisons of gene expression levels, staining intensities and survival times were performed using paired or unpaired t-test, when appropriate. Primary tumor growth was compared using the nonparametric Mann-Whitney-Wilcoxon test, while Chi-square test was used for metastasis frequency data analysis.
